# The role of gut microbiota in the effects of maternal obesity during pregnancy on offspring metabolism

**DOI:** 10.1042/BSR20171234

**Published:** 2018-04-13

**Authors:** Liyuan Zhou, Xinhua Xiao

**Affiliations:** Department of Endocrinology, Key Laboratory of Endocrinology, Ministry of Health, Peking Union Medical College Hospital, Diabetes Research Center of Chinese Academy of Medical Sciences and Peking Union Medical College, Beijing 100730, China

**Keywords:** gut microbiota, maternal obesity, metabolism, offspring, pregnancy

## Abstract

Obesity is considered a global epidemic. Specifically, obesity during pregnancy programs an increased risk of the offspring developing metabolic disorders in addition to the adverse effects on the mother *per se*. Large numbers of human and animal studies have demonstrated that the gut microbiota plays a pivotal role in obesity and metabolic diseases. Similarly, maternal obesity during pregnancy is associated with alterations in the composition and diversity of the intestine microbial community. Recently, the microbiota in the placenta, amniotic fluid, and meconium in healthy gestations has been investigated, and the results supported the “in utero colonization hypothesis” and challenged the traditional “sterile womb” that has been acknowledged worldwide for more than a century. Thus, the offspring microbiota, which is crucial for the immune and metabolic function and further health in the offspring, might be established prior to birth. As a detrimental intrauterine environment, maternal obesity influences the microbial colonization and increases the risk of metabolic diseases in offspring. This review discusses the role of the microbiota in the impact of maternal obesity during pregnancy on offspring metabolism and further analyzes related probiotic or prebiotic interventions to prevent and treat obesity and metabolic diseases.

## Introduction

Obesity has become an epidemic disease and is associated with tremendous health and economic burden all over the world. The World Health Organization (WHO) newly announced that there were more than 1.9 billion overweight adults worldwide in 2014, and of these, over 600 million were obese. Furthermore, over 300 million women suffer from obesity (WHO Obesity and overweight Fact Sheet No: 311, updated June 2016). A recent study on the global prevalence of obesity showed that 50% of women of childbearing ages and 20–25% of pregnant women in Europe were affected by overweight or obesity [1]. As a major risk factor for metabolic syndrome, diabetes and cardiovascular disease, obesity could lead to various metabolic disorders, including insulin resistance, hyperglycemia, hyperlipidemia, and hypertension. Obesity in women of reproductive age not only gives rise to adverse effects on the mother *per se*, but also affects the intrauterine environment. Barker first discovered that men with lower birth weights had higher death rates from ischemic heart disease [2] and thus put forward the “the thrifty phenotype hypothesis” [3]. He suggested that maternal under-nutrition during pregnancy and early post-partum would program offspring to adapt to this thrifty environment and develop various dysfunctions. Thereafter, the Developmental Origins of Health and Disease (DOHaD) hypothesis was brought forward in the last decade [4,5], which proposed that an adverse developmental environment *in utero* and early postnatal life negatively influences long-term health and increases the risk of developing obesity [6], diabetes [7], cardiovascular disease [8], and other chronic diseases. Hence, maternal obesity during pregnancy programs an increased risk of the offspring developing metabolic disturbances. Although obesity-susceptibility genes inherited from the mother may partially explain this phenomenon [9,10], substantial studies have demonstrated that epigenetics might play a role in the effects of maternal health on offspring [11–13]. However, the specific epigenetic regulation mechanism is still unclear as of yet.

**Figure 1:**
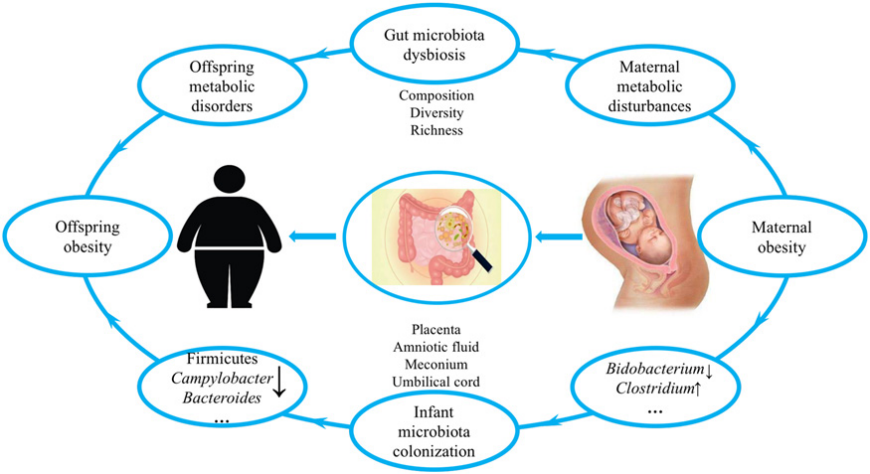
Overview of the role of gut microbiota in effects of maternal obesity during pregnancy on offspring metabolism [15,16,93,97]

Recently, a growing number of human studies [14,15] and animal experiments [16–18] put forward the hypothesis that the gut microbiota may be identified as a novel factor that plays a significant role in maternal obesity and associated metabolic risks in offspring. During the last few decades, the gut microbiota has become a focus of medical research and has been shown to be intertwined with various diseases, such as obesity [19], diabetes [20], autoimmune diseases [21], cancer [22], and central nervous system diseases [23]. As containing the second genome in the human body, the gut microbiota has important physiological functions such as helping the host absorb fats and fat-soluble vitamins in their diet and digest complex carbohydrates and plant polysaccharides and participating in bile acid-related metabolism. In addition, gut microbiota also plays important roles in maintaining the intestinal epithelial barrier, regulating intestinal permeability, promoting maturation of the enteric nervous system, developing innate immunity, and regulating adaptive immunity [24–27]. In healthy people, the commensal microbiota in the gut is in a balanced symbiosis with the host. However, the equilibrium of this symbiotic relationship is susceptible to the host’s diet, lifestyle, and antibiotics [28]. Substantial human and animal evidence has demonstrated that the composition and diversity of the gut microbiota are altered in obese development [19,29,30]. Some studies have shown a typical increase in the number of Firmicutes and a decrease in the number of Bacteroidetes in obese compared with those in lean individuals and mice [31]. However, some have reported an inversely altered ratio of Firmicutes:Bacteroidetes in obesity [32]. The bacteria that is present or absent and contributes to the development of obesity still has yet to be elucidated. Although the infant gut microbiota used to be widely accepted to be established and colonized postpartum [33,34], in recent years, emerging studies have discovered that the microbiota exists in the placenta, amniotic fluid, umbilical cord blood, and meconium, which strongly indicates that the offspring’s microbiome may be transmitted from the mother prior to birth, and maternal microorganisms are likely to play a vital role in the establishment of the offspring microbiome [35–38]. Hence, maternal obesity during pregnancy is accompanied with gut microbiota dysbiosis with a simultaneous development of metabolic disorders, which could affect microbiota transmission from the mother to offspring and further result in offspring metabolic disturbances (Figure 1). In this review, we discuss the role of the gut microbiota in the impact of maternal obesity during pregnancy on offspring metabolic health and further explore related strategies, including probiotic and prebiotic strategies, to prevent and treat obesity and obesity-related diseases.

## Impact of obesity during pregnancy on maternal and offspring metabolism

With changes in diet and lifestyle, numerous evidence has demonstrated that the prevalence of obesity in women of childbearing ages is still increasing rapidly [39–41]. Maternal obesity during pregnancy, resulting in a poor intrauterine environment, has adverse outcomes for both the mother and child [42].

Obesity in pregnancy not only affects maternal metabolism, but also leads to detrimental pregnancy outcomes. A population-based prospective cohort study in the Netherlands detected that maternal obesity during pregnancy increased the risks of gestational diabetes, gestational hypertension, preeclampsia, and caesarean section compared to pregnancy with normal weight [43]. Similarly, another systematic review of 22 reviews also showed that gestational diabetes, pre-eclampsia, gestational hypertension, depression, instrumental and caesarean delivery, and surgical site infection were strongly linked with maternal obesity in contrast to women with normal body mass index (BMI). Obesity in pregnancy is also associated with a greater risk of adverse pregnancy consequences, including large-for-gestational age (LGA) babies, preterm birth, fetal defects, congenital anomalies, and perinatal death [44].

Moreover, animal studies also demonstrated that an obesogenic high-fat diet during pregnancy caused maternal hyperinsulinemia and decreased insulin sensitivity [45]. Mina and colleagues also used a high-fat diet prior to and throughout pregnancy and lactation to create obese animal models and found that body fat and plasma corticosterone levels were both elevated in high-fat diet–fed dams [46].

In addition to the impact on maternal health, a body of studies demonstrated that overnutrition during pregnancy increased the susceptibility to metabolic diseases in offspring [42,47]. In the Helsinki Birth Cohort Study, Eriksson et al. [48] indicated that maternal pregnancy BMI was positively associated with offspring health outcomes later in life, particularly cardiovascular disease and type 2 diabetes. Similarly, in another population-based prospective cohort study, Gaillard and his colleagues [49] reported that compared with children from normal-weight mothers, those from obese mothers during pregnancy had elevated risks of childhood obesity and adverse cardiometabolic outcomes, including total body and abdominal fat mass, systolic blood pressure, insulin levels, and high-density lipoprotein cholesterol (HDL-c) levels.

Furthermore, substantial animal studies performed to verify this phenomenon and the concrete mechanisms are emerging. Maternal high-fat diet (HFD) during pregnancy leads to a poor fetal intrauterine developmental environment, which predisposes offspring to develop metabolic disorders. Bringhenti et al. [50] and Wankhade et al. [51] demonstrated that offspring born to HFD-fed dams from weaning to end of lactation, which mimics human continuous high-fat food intake, gained more body weight and adiposity than their control diet-fed littermates. In contrast, Bringhenti et al. showed that offspring from HFD dams had hypercholesterolemia, hypertriacylglycerolemia, and hyperinsulinemia as well as a higher level of leptin with reduced levels of adiponectin. Bringhenti et al. analyzed the mechanisms that led to metabolic abnormalities in the adult offspring through examining islet structure and function and observed increases in the masses of the islet, beta cells, and alpha cells, along with migration of the alpha cells from the edge to the core of the islet and down-regulation of insulin receptor substrate (IRS1), phosphatidylinositide 3-kinase (PI3k), pancreatic and duodenal homeobox 1 (Pdx1), and glucose transporter 2 (Glut2) protein expressions. They suggested that maternal HFD was responsible for impairments in the insulin signaling pathway and altered islet structure in adult offspring. However, Wankhade et al. showed enhanced predisposition to steatohepatitis in offspring born to HFD dams, accompanied by decreased α-diversity in gut microbiota profiles. In addition to the effects of maternal obesity on offspring peripheral metabolic health, a number of studies have shown that central metabolism alterations should also not be ignored. A recent study of a porcine model with the use of positron emission tomography (PET) showed that maternal HFD led to higher brain glucose metabolism and brain insulin receptors (IRβs) at birth in offspring followed by a long-term decrease in brain glucose uptake and glucose transporter 4 (GLUT4) and IRβ expression in minipigs, which may increase the predisposition to metabolic-neurodegenerative diseases in offspring [52].

Hence, obesity during pregnancy serves as a detrimental developmental environment that increases the risk of the development of metabolic diseases in both the mother and offspring. However, the specific mechanisms are not entirely understood. The relationship between the gut microbiota and obesity has been increasingly studied. Meanwhile, the significant role that the gut microbiota in newborns and infants plays in health and development has become increasingly clear. Therefore, the gut microbiota might be a critical factor in the explanation of the phenomenon put forth by the DOHaD Hypothesis.

## Maternal obesity during pregnancy and alterations in gut microbiota

In the context of normal pregnancy, bodies undergo substantial changes including immunological adaption and hormonal and metabolic alterations to support the growth and development of the fetus [53]. Similar to the symptoms of metabolic syndrome, increased adiposity, reduced insulin sensitivity, and elevated levels of proinflammatory cytokines in circulation are all common but protective during a healthy pregnancy [54,55]. The commensal microbial community in the human gastrointestinal tract plays crucial roles in the immune response and metabolic homeostasis and, thus, in normal pregnancy [56,57]. In order to explore the role of gut microbiota during pregnancy, Koren et al. [58] analyzed the fecal microbiota of 91 pregnant women from the first to third trimesters with the use of 16S rRNA gene sequences (V1–V2 region) and found that while the microbial diversity was analogous to that of the Human Microbiome Project’s (HMP) normal controls during early pregnancy, significant changes in the microbial community occurred in the third trimester. Specifically, the abundance of Proteobacteria and Actinobacteria was increased but their phylogenetic diversity was reduced. In accordance with the changes in Proteobacteria, which is correlated with inflammation [59], levels of proinflammatory cytokines IFN-γ, IL2, IL6, and TNF-α were significantly increased from the first to third trimester. Furthermore, transference of the microbiota from healthy pregnancies in the third trimester to germ-free mice also resulted in mice with increased adiposity, reduced insulin sensitivity, and a chronic inflammatory condition compared with mice that received microbiota from those in the first trimester [58]. Therefore, the gut microbial community plays a critical role in maternal metabolic adaptations and supports the growth and development of the fetus during normal pregnancy. However, another study done by DiGiulio et al. [60] showed outcomes completely different from Koren’s. They analyzed the microbiota from a case–control study including 49 pregnant women, 15 of whom delivered preterm, with the use of 16S rRNA gene sequences (V3–V5 region). From 40 of these women, they characterized the bacterial taxonomic composition of 3767 specimens collected prospectively and weekly during gestation from the vagina, distal gut, saliva, and teeth/gums rather than a single time point from each of the first and third trimesters as in Koren’s study. Consequently, DiGillio et al. demonstrated that the composition and diversity of the gut microbiota in pregnant women were stable. The distinct differences between Koren’s and DiGiulio’s study outcomes may be partly attributed to heterogeneity in genetics, age, ethnicity, lifestyle, BMI, and gestational age of the participants [61]. Specifically, in Koren’s study, 16 of the women took probiotic supplements during pregnancy, and seven used antibiotics that could greatly influence the composition and diversity of the gut microbiota. Preterm delivery and different 16S rRNA gene sequencing regions might be another two major influencing factors. Furthermore, more studies are needed to clarify the changes in the gut microbiota during normal pregnancy and its effects on fetal and maternal development.

Substantial evidence has demonstrated that the diversity and function of the gut microbiota changes in nonpregnant obesity. To be exact, compared with that observed in lean subjects, the microbial diversity is lower and the ratio of Firmicutes to Bacteroidetes is altered, although controversial, in obese subjects [62–64]. Similarly, changes in the gut microbiota occur in overweight and obese pregnant women when compared with that in normal-weight pregnant women. Collado et al. [65] first compared the composition of the gut microbiota assessed by fluorescent *in situ* hybridization coupled with flow cytometry (FCM-FISH) and by quantitative real-time polymerase chain reaction (qPCR) in overweight and normal-weight pregnant women from a prospective follow-up study and found significant alterations in the gut microbial community between the two groups, of which *Bacteroides* and *Staphylococcus* were the most significantly increased in overweight and obese women. However, another subsequent observational study of 50 pregnant women in Spain analyzed the composition of the gut microbiota by qPCR and showed that *Enterobacteriaceae, Escherichia coli*, and *Staphylococcus* numbers were increased but *Bifidobacterium* and *Bacteroides* abundances were significantly reduced in overweight and obese pregnant women, which is not consistent with the previous study. The ratio of *Bifidobacterium* to *Clostridium coccoides* was significantly lower during pregnancy in overweight and obese women than in normal-weight women. These researchers also analyzed the relationship between gut microbiota composition and biochemical parameters. Increased total bacteria and *Staphylococcus* numbers were positively related to increased plasma cholesterol levels. Decreased *Bacteroides* numbers were associated with lower HDL-cholesterol and folic acid levels and higher TAG levels. These associations could be explained by the generation of different short-chain free acids (SCFAs) and regulation of the host gene expression to influence lipid metabolism [66–68]. Lower *Bifidobacterium* numbers were related to reduced folic acid levels, which may result from the ability of this genus to synthesize and secrete folates in the intestinal environment, providing a complementary endogenous source of this vitamin [69]. Increased *Enterobacteriaceae* and *E. coli* numbers were associated with increased ferritin and reduced transferrin [70]. A decrease in transferrin and an increase in ferritin were associated with a decrease in antibacterial activity of serum against Enterobacteria [71]. Thus, distinct changes of the gut microbiota occur in overweight and obese women during pregnancy. These discrepant findings require further confirmation and may be explained by heterogeneity in the gestational age, genetics, dietary intake, and supplementation of the participants and especially by different assessing methods.

In addition, emerging animal studies verified the relationship between maternal obesity and changes in the gut microbiota. In female Sprague–Dawley rats, a high-fat/sucrose diet-induced obesity group had higher blood glucose, plasma insulin, and leptin concentrations and lower peptide-YY (PYY) levels than the lean group. Meanwhile, the composition of the fecal microbiota was altered, with reduced fecal *Bidobacterium* spp*.* numbers and increased number of *Clostridium* clusters XI and I [16]. The phenomenon that gut microbiota shifted physiologically during pregnancy in order to adapt the mother to pregnancy and facilitate optimal fetal growth and development was simultaneously demonstrated in animal study. A recent animal study suggested that there were 26 genera of gut microbiota found to be significantly different in HFD pregnant mice from those in normal diet mice. Of these, 11 genera were also identified to be significantly different between nonpregnant mice fed normal diet and nonpregnant HFD–fed mice, and another 15 genera were independently associated with the pregnant state. Furthermore, these shifts in the gut microbiota could predict the changes in lipid metabolism, glycolysis, and gluconeogenic metabolic pathways [72].

Alterations in the gut microbial community are an attempt to adapt the mother to pregnancy, contribute to the function of the placenta, and promote fetal growth and development in the physiologically pregnant state. During obese pregnancy, as showed above, altered gut microbiota will lead to metabolic disturbances in mother by affecting the production of SCFAs, participating in the metabolism of other substances and regulating gene expression in host, which indirectly could affect the infant’s development and the establishment of its microbiota. Although the altered composition of the gut microbiota is still controversial among different studies, and the concrete mechanisms modulating maternal gut microbial alterations are currently unclear, overweight and obesity during pregnancy result in obvious changes in microbiota composition and concomitant metabolic disorders in the mother.

## Establishment of the infant microbial community

The healthy adult human gastrointestinal tract is colonized by more than 10^14^ bacteria with over 1000 prevalent species, which include 100 times more genes than the human genome [73–75]. The first colonization of gut microbiota in early life is a critical window for development and further health and plays a significant role in programming immune development and metabolic function [35]. For more than a century, the intrauterine environment has been accepted to be sterile, and the microbiota of a neonate has been thought to be colonized during birth and in postnatal life, although there was some early controversy [33,76]. However, the recent “in utero colonization hypothesis” challenges the idea of the traditional “sterile womb” [77]. In the last few years, a multitude of emerging human and animal studies have indicated that the infant microbiome is probably established before birth as specific microbiota and microbial DNA were detected in the placenta, amniotic fluid, umbilical cord, and infant meconium during normal pregnancy with the use of modern sequencing technologies, indicating that the colonization of the infant microbiota was initiated *in utero* [38,78,79].

In a study of 21 healthy term neonates, a number of bacteria were detected in the meconium first through traditional culture, among which genera *Enterococcus fecalis* and *Staphylococcus* were the most predominant [79]. Then, another study collected the meconium of 23 newborns, including 9 neonates from healthy mothers, and 14 born to mothers with diabetes or pre-diabetes. Using 16S rRNA sequencing to profile the meconium microbiota, the study showed that the meconium microbiota of newborns born to healthy mothers was significantly different from adult stool, including a lower proportion of Bacteroidetes and Firmicutes that are the predominant phyla in adult stool samples and a higher proportion of Proteobacteria [80]. This observation suggested that the bacterial composition of the meconium was a proxy for the gut microbiota *in utero* and supports the theory of microbiota colonization prior to birth. Subsequently, Collado et al. [38] analyzed the microbiota in the maternal placenta, amniotic fluid, and meconium of 15 caesarean-section at-full-term mother–infant pairs and proposed that a unique microbiota exists in the placenta and amniotic fluid, characterized by low abundance, low diversity, and predominant Proteobacteria. The meconium also shared some similarities with the microbial communities in the placenta and amniotic fluid, which could be explained by the fetus swallowing the amniotic fluid. Our latest study first compared the placental microbiota between macrosomic newborns and newborns with normal birth weight and demonstrated that the microbiota in the placenta of normal neonates was indeed significantly different from that in the macrosomic placenta [81].

However, the exact mechanism of maternal–fetal microbial transfer is still unclear and several hypotheses have been put forward. Interestingly, Perez et al. [82] indicated that bacterial translocation of the intestinal mucosa was significantly increased in pregnant mice compared with that in nonpregnant animals in 2007. Then Jiménez et al. [79] orally infused genetically labeled *E. fecium* strain to pregnant mice and reported that the strain could be detected in the meconium of the infused mice but not in the meconium of the noninfused control group in 2008. Thus, the microbial community in the maternal gastrointestinal tract influences the establishment of the fetal microbiota in the meconium. In another words, the meconium microbial community might originate from the maternal intestine tract. Differently, after analyzing the placental microbiota from 320 subjects, Aagaard et al. [36] suggested that the placental microbiota mainly included microbiota from Firmicutes, Tenericutes, Proteobacteria, Bacteroidetes, and Fusobacteria phyla and were most similar to the human oral microbiota. However, they compared the placental microbiota to the microbiota of other nonpregnant human body sites, including the oral, skin, airway, vaginal, and gut microbiota, and thus, the results were not very convincing. Another more recent study done by Gomez-Arango et al. [83] agreed with Aagaard and demonstrated that the placental microbiota exhibited a higher similarity to the pregnant oral microbiota. What is more, the gut, oral, and placental microbiota they detected by 16S rRNA sequencing were from the same pregnant women [83]. The vaginal microbiota might be another origin of the fetal microbiota by virtue of its proximity to the intrauterine environment. *Lactobacilli* is the predominant microbiota in the vagina during pregnancy [60,84]. Although *Lactobacilli* are indeed found in placental membranes [85], the microbiota *in utero* is diverse, as mentioned previously. Thus, the vagina is at least not the only colonization pathway of the fetal microbiome. The exact origin of the intrauterine microbiota is still unclear and requires more research.

Previous data showed that bacteria could be detected *in utero* only in cases of intrauterine infection and preterm delivery, and the intrauterine environment was sterile during healthy pregnancy based on microscopy and bacterial culture techniques [33,86,87]. With the development of modern sequencing methods, emerging evidence has demonstrated that microbial communities do exist in the placenta, amniotic fluid, and meconium. However, Perez-Muñoz et al. [77] described the weakness of the evidence supporting the “*in utero* colonization hypothesis”, including a lack of appropriate controls for contamination, failure of detection approaches to detect low-abundance microbial communities, and no evidence of bacterial viability. Therefore, more studies are needed to reveal the time and mechanism of vertical microbiota transmission and colonization in offspring.

## The effects of maternal obesity on the offspring gut microbiota

Both our own studies and other studies have shown that maternal HFD could program a significantly increased predisposition to obesity and metabolic disorders in offspring [46,88–90]. As mentioned above, obesity of pregnant mothers alters the abundance and diversity of the gut microbiota and, thus, as a detrimental intrauterine developmental environment, may influence the establishment of the microbial community in offspring. Large numbers of studies have verified that maternal obesity is associated with gut microbiota dysbiosis in offspring. In a mouse study done by Wankhade et al., a significantly lower α-diversity of the fecal microbial community was identified in offspring of mice with maternal HFD, which was concomitant with a greater weight gain, fatty liver, and increased proinflammatory hepatic transcriptome in the offspring [51]. Furthermore, Bruce-Keller et al. [93] transplanted gut microbiota from mice fed a HFD or control diet for 3 months to mothers. The β-diversity of the gut microbiota in both weaned female and weaned male offspring from the HFD-fed dams was significantly lower than that from the control diet dams, among which the numbers of Firmicutes phylum were reduced. The Firmicutes phylum have the ability to produce butyrate, a main energy source for intestinal epithelium, playing a critical role in maintaining the integrity of the intestinal epithelial barrier [91,92] and associated with neurobehavioral disorder [93]. Studies have shown that abnormal SCFAs produced by gut microbiota dysbiosis could increase host energy extraction from the diet and influence the levels of satiety hormone, which could lead to altered food intake and modify immune cells in the intestinal tract, further influencing metabolic health in the mother and vertically transmitting to the offspring [94–96]. In addition to the effects on intestinal microbiota establishment in early life, in a non-human primate model, Ma et al. [97] demonstrated that maternal HFD compared with a maternal control diet persistently reduced the abundance of *Campylobacter* until the primates were juveniles.

Moreover, human studies have also consistently indicated that maternal obesity and overnutrition alter the gut microbiota in offspring. In a prospective cohort study done by Chu et al. [15], 163 women in the early third trimester or intrapartum were enrolled and divided into a high-fat group and control group according to a dietary questionnaire covering the past month. The meconium and stool of neonates at delivery and 6 weeks of age were collected and subjected to 16S rRNA gene sequencing. A significant reduction in *Bacteroides* in the neonatal intestinal microbiota from HFD mothers during gestation was found that persisted to 6 weeks of age [15]. Similarly, another study analyzed the gut microbiota of 77 children born to obese or normal weight mothers and showed that the numbers of *Parabacteroides* spp*.* and *Oscillibacter* spp*.* were higher in children born to obese mother than in those born to normal weight mothers from a higher socioeconomic status; additionally, the numbers of *Blautia* spp*.* and *Eubacterium* spp*.* were lower, of which *Eubacteriaceae, Oscillibacter*, and *Blautia* have been found to be associated with diet and obesity in previous studies [98,99]. Hence, the increased risk of obesity for children with an obese mother could be explained partially by the transmission of maternal obesogenic intestinal microbes [17]. The changes in the gut microbiota in offspring born to obese mother have still been controversial in recent studies. The differences may be due to heterogeneity in BMI, mode of delivery, feeding methods in early life, and use of antibiotics at delivery. More studies that control for these confounding factors are needed to clarify the specific changes in offspring.

Both animal and human studies have demonstrated that changes in the diversity and abundance of the intestinal microbial community in mothers associated with maternal HFD and obesity were linked with gut microbiota alterations in the offspring in early and later life. In addition, the metabolic health of the offspring was impaired. Thus, the gut microbiota may be a crucial pathogenic mechanism in the maternal obesity programing of an increased susceptibility to obesity and metabolic disorders in offspring. Improvement in the maternal gut microbiota dysbiosis might also promote offspring metabolic health and maintain the balance of the intestinal microbial community.

## Intervention with probiotics and prebiotics in the mother

### Probiotics

Probiotics have been defined by an international consensus panel as “live microorganisms that, when administered in adequate amounts, confer a health benefit on the host” [100]. Certain specific probiotics can be used to decrease the risk of the development of diseases correlated with increased intestinal permeability, abnormal gut microbiota composition, or impaired immunological or metabolic balance [101]. An increasing number of human and animal studies have shed light on the beneficial effects of probiotic supplements on obesity and metabolic disturbances. A probiotic formulation could ameliorate obesity and obesity-related metabolic disorders by virtue of its action on the composition of the gut microbiota, intestinal barrier integrity, and related metabolites [102–104]. Intervening with probiotics during pregnancy could be the optimum time to reduce the risk of obesity and metabolic disturbances in both the mother and offspring.

A randomized controlled trial (RCT) done in Finland recruited 256 pregnant women without metabolic disorders during their first trimester. Participants were randomized into a control group, dietary counselling intervention group, or dietary counselling with probiotics (*Lactobacillus rhamnosus* GG and *Bifidobacterium lactis* Bb12) intervention group from the first trimester of pregnancy to 6 months after delivery [105–107]. In the dietary counselling with probiotics group, the fasting blood glucose concentrations were the lowest, and glucose tolerance was better during pregnancy and through the 12-month postpartum period. Furthermore, the frequency of gestational diabetes mellitus (GDM) decreased from 34% in the control group to 13% in the group that received dietary counselling and probiotics. Meanwhile, probiotics-supplemented dietary counselling reduced the risk of central obesity in women at 6 months postpartum. This study suggested that probiotic supplements might be a novel management tool for the prevention and treatment of obesity. In addition, another similar RCT study in Iran with 64 pregnant women with GDM randomized to receive probiotic supplement (*Lactobacillus acidophilus* LA-5, *Bifidobacterium* BB-12, *Streptococcus thermophilus* STY-31, and *Lactobacillus delbrueckii bulgaricus* LBY-27) or placebo for 8 weeks also found that the probiotic supplements significantly reduced gestational weight gain and fasting blood glucose and improved insulin sensitivity [108]. Wickens et al. [109] also indicated that probiotic *Lactobacillus rhamnosus* HN001 supplementation from 14 to 16 weeks’ gestation in women with a history of atopic disease may reduce GDM prevalence from 6.5% (95% CI 3.5, 10.9) in the placebo group to 2.1% (95% CI 0.6, 5.2). However, neither of the above RCT studies examined the effects of maternal probiotic supplements on the metabolic health of the offspring in later life. A follow-up study from birth to 10 years of age with 159 pregnant women in Finland aimed to identify the influences of probiotic (*Lactobacillus rhamnosus* GG, ATCC 53103) intervention from 4 weeks before delivery to 6 months postnatally on the development of overweight and obesity. Prenatal probiotic supplements restrained excessive weight gain during early life (before 12–24 months) and may have altered the growth pattern in offspring. Gut microbiota modification of probiotics in early life might adjust the energy homeostasis and thus influence the body weight gain and prevent obesity [110].

Rather than examining the effects of probiotics in a normal-weight pregnancy, a double-blind, placebo-controlled, randomized trial in Ireland explored the impact of probiotic intake on obese pregnancy. They recruited 179 pregnant women whose BMI was from 30.0 to 39.9 at early pregnancy and then divided them randomly into groups that received probiotic (*Lactobacillus salivarius* UCC118) or placebo capsules from 24 to 28 weeks of gestation, and 138 women completed the study. This probiotic strain was chosen by virtue of its existence in the intestinal tract of normal people and its impact on immune reactions [111]. The results showed that the 4-week probiotic capsule intervention during pregnancy had no effect on maternal fasting glucose, the prevalence of impaired glucose tolerance (IGT), infant birth weight or other metabolic profiles or pregnancy outcomes. After adjustments for the use of antibiotics and compliance, the results were still the same [112]. However, there were only 9 cases of GDM and 15 cases of IGT in this study, and thus, the statistical power was limited. Furthermore, the probiotic intervention was only performed for 4 weeks, and the bacteria strain chosen may not have been optimal for obese women during pregnancy as it has merely been studied in the healthy population.

The optimal bacteria strains of probiotics and the concrete mechanisms for their positive effects are still unclear. Current literature is limited to studies with low statistical power and a general lack of consistency in their population characteristics and probiotic strains. We look forward to the results of another RCT study exploring probiotics to prevent gestational diabetes mellitus in overweight and obese women in Australia, which used the same probiotic strains as the first study in Finland. This SPRING trial may demonstrate a role of probiotics in reducing the prevalence of GDM in high-risk pregnant women [113].

### Prebiotics

Prebiotics were first defined in 1995 as “a nondigestible food ingredient that beneficially affects the host by selectively stimulating the growth and/or activity of one or a limited number of bacteria already resident in the colon” [114]. After that, the concept of prebiotic was reaffirmed several times, and the latest definition was updated by the International Scientific Association for Probiotics and Prebiotics in December 2016 as “a substrate that is selectively utilized by host microorganisms conferring a health benefit.” This concept indicates that prebiotics include not merely carbohydrates but also substances other than food and that prebiotics could manipulate the microbiota in regions other than in the intestinal tract [115]. Prebiotics are defined to be beneficial to human well-being in terms of both the modulation of the microbiota and the production of metabolites. Prebiotics contribute to health by increasing the growth of *Bifidobacteria* and *Lactobacilli* and producing SCFAs, which are considered to be beneficial to human well-being by improving the integrity of the gut barrier, modulating the host immune reaction, and decreasing the abundance of pathogenic bacteria, such as Clostridia [116]. Substantial evidence has demonstrated that prebiotic intake could reduce the risk of developing diarrhea [117], inflammatory bowel disease [118], colon cancer [119], cardiovascular disease [120], obesity [121], metabolic disturbances [122], and other diseases and increase the uptake and bioavailability of calcium and magnesium [123].

Studies on the effects of maternal prebiotic intake during pregnancy on offspring are listed in Table 1. In light of the impact of prebiotic intervention during pregnancy on the metabolic health of mothers and their offspring, several animal studies have been done to shed light on this phenomenon [16,124–126]. First, Maurer et al. [124] explored the influences of a maternal high-prebiotic fiber diet (21.6%) on the expression of genes regulating glucose and lipid metabolism and satiety hormones in offspring. In offspring from dams fed the high-prebiotic fiber diet, the weights of the small intestine and colon were increased significantly at 14 and 21 days; the level of plasma amylin was lower at 7, 14, and 21 days; the expression of intestinal glucose transporters 5 (GLUT5) and Na^+^-dependent glucose/galactose transporter 1 (SGLT 1) was up-regulated; and the level of glucagon-like peptide-1 (GLP-1) was higher at 21 days than in offspring born to control diet-fed dams. Thus, a maternal high-prebiotic fiber diet might program the development of the intestinal tract and the expression of satiety hormones and genes associated with glucose metabolism in offspring in early life, which could influence the long-term obesity risk. Similarly, Hallam et al. [125] suggested that offspring of dams fed a high-prebiotic fiber diet (21.6%) had a lower plasma lipopolysaccharide level and elevated *Bifidobacteria* in the gut microbiota until 22 weeks with the use of an animal model and experimental design analogous to Maurer’s. These results indicated that the maternal high-prebiotic fiber diet contributed to offspring health by modulating the composition of the gut microbiota. Meanwhile, Hallam et al. [126] switched offspring from a control diet to a high-fat/sucrose diet for 8 weeks at 14.5 weeks of age and demonstrated that after a high energy challenge, the weight, percentage of fat mass, liver TAG content, and plasma nonesterified fatty acid (NEFA) levels were lower in female offspring of dams fed the high-prebiotic fiber diet. Therefore, maternal prebiotic intake could reduce the risk of obesity and improve long-term metabolic health in offspring. However, only one of the above studies measured changes in the gut microbiota in offspring after prebiotic intervention. Another recent study assessed the impact of prebiotic intervention during pregnancy and lactation on the composition of the maternal gut microbiota and suggested that an intervention with 10% oligofructose in high-fat/sucrose diet-induced obese rats during pregnancy and lactation could improve the maternally altered gut microbiota with a higher relative abundance of *Bifidobacterium* spp., *Bacteroides/Prevotella* spp., and *Clostridium* cluster I as well as reduce the energy intake and gestational weight gain compared with those in the high-fat/sucrose diet group. In addition, the risk of obesity and the metabolic profiles of their offspring were both ameliorated [16]. These observations suggested that early prebiotic intervention in the mother benefited both the maternal and offspring metabolic health and was of great clinical significance. More studies are needed to verify these findings and further clarify the concrete mechanism of the positive effects of prebiotics.

**Table 1:** Evidence that maternal prebiotic exposures during pregnancy are associated with programming offspring health

Maternal prebiotic exposures	Animal model	Effects on offspring	Reference
Galacto-oligosaccharide/inulin mixture	Balb/c mice	Prevents food allergies	[127]
Specific mixture of short-chain galacto- and long-chain fructo-oligosaccharides	Balb/c and C57BL/6 mice	Reduces symptoms of allergic asthma	[128]
Fructo-oligosaccharide	NC/Nga mice	Diminishes the severity of atopic dermatitis-like skin lesions	[129]
Oligofructose	Sprague–Dawley rat	Reduces obesity risk	[16]
Oligofructose and inulin	Wistar rat	Decreases fat mass	[125]
Inulin and oligofructose	Wistar rat	Regulates expression of circulating metabolism associated hormones and genes	[124]
Oligofructose-enriched inulin	Sprague-Dawley rat	Improves bone microarchitecture	[130]
Caprine milk oligosaccharides	C57BL/6 mice	Improves the development	[131]
Galacto-oligosaccharides-inulin	Balb/cj mice	Enhances intestinal and muscle mass growth	[132]
Short-chain fructo-oligosaccharide	Sow	Strengths gut defenses and immune response	[133]

## Conclusion

In summary, the gut microbiota might be the key programming factor in maternal obesity increasing the risk of the development of metabolic disorders in offspring. However, the mechanisms remain unclear. In addition to direct vertical transmission of microbes, microbial dysbiosis influences bile acid metabolism in the host body and changes the metabolites of the microbiota, such as SCFAs, which play important roles in host metabolism and immunity to affect metabolic health in both the mother and offspring [134]. In addition, some scholars have suggested that the microbiota could impact a set of epigenetic factors, including DNA methylation, histone modification and noncoding RNAs, which will further change metabolism-related gene expression in the host [135]. Thus, gut microbiota dysbiosis in obese mothers leads to multiple downstream effects on immune and metabolic functions and further health in offspring.

There have been a few studies that have suggested that probiotics and prebiotics positively modulate the gut microbiota and their metabolites, restrain weight gain, reduce the frequency of gestational diabetes mellitus, and improve blood glucose, insulin sensitivity and glucose metabolism-associated gene expression. Changing the composition of the gut microbiota specifically through the use of probiotics and prebiotics during pregnancy could become a new strategy for reducing the risk of metabolic disorders in both the mother and offspring. Consequently, there is an urgent need for large, well-designed randomized controlled clinical trials to further dissect the use of probiotics and prebiotics as a prevention strategy, in addition to mechanistic studies clarifying potential mechanisms of action in humans.

## Abbreviations

BMI: body mass index
HDL-c: high-density lipoprotein cholesterol
HFD: high-fat diet
IFN-γ: interferon-γ
IGT: impaired glucose tolerance
IL2: interleukin 2
IL6: interleukin 6
IRβ: insulin receptor
LGA: large-for-gestational age
SCFA: short-chain free acid
TNF-α: tumor necrosis factor-α
